# The complete chloroplast genomes and phylogenetic analysis of *Exbucklandia longipetala* and *Exbucklandia populnea* (Hamamelidaceae)

**DOI:** 10.1080/23802359.2024.2406933

**Published:** 2024-09-30

**Authors:** Shuang Xiong, Fuqin Zhou, Shidong Wang, Yuan Huang

**Affiliations:** School of Life Sciences, Yunnan Normal University, Kunming, People’s Republic of China

**Keywords:** Chloroplast genome, phylogenetic relationships, *Exbucklandia*, Hamamelidaceae

## Abstract

*Exbucklandia longipetala* and *Exbucklandia populnea* are two evergreen trees of the genus *Exbucklandia* in the family Hamamelidaceae. In this study, the complete chloroplast genomes of *E. longipetala* and *E. populnea* were sequenced, assembled, and annotated. The total lengths of the chloroplast genomes were 160,723 bp and 160,744 bp, respectively, and both had a GC content of 38.1%. The complete chloroplast genomes of these two species had typical quadripartite structures: LSC region (88,972 bp and 88,989 bp), SSC region (18,907 bp and 18,911 bp) and a pair of inverted repeats both of 26,422 bp. Both species contained 114 unique genes, including 80 protein-coding genes, 30 tRNA genes, and 4 rRNA genes. Phylogenetic analysis indicated that *E. longipetala* and *E. populnea* are sister species to each other. Our results provide useful genetic resources for further studies on the origin and evolution of Hamamelidaceae.

## Introduction

**Figure 1.**
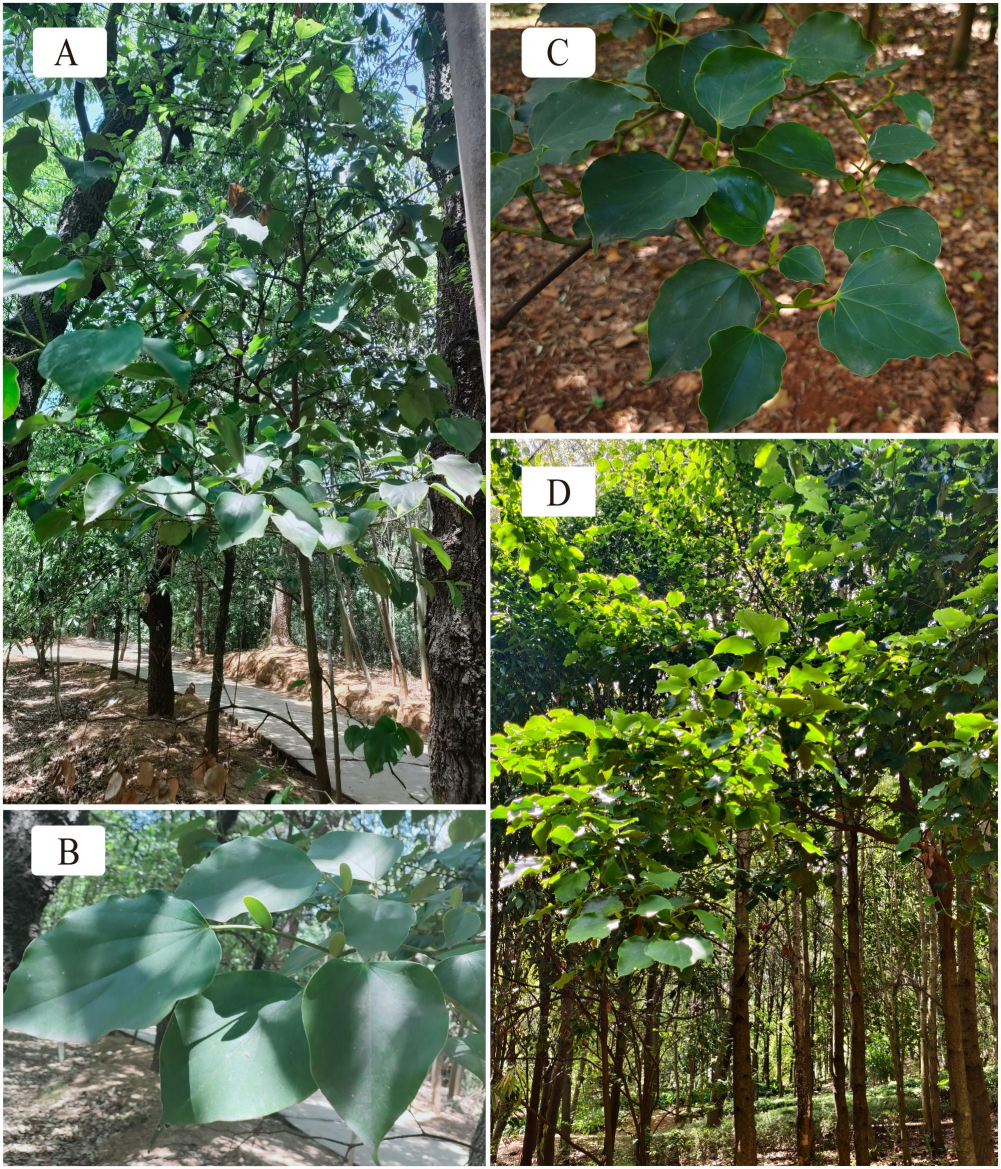
Plant individuals of *E. longipetala* and *E. populnea*. (A) Whole plant of *E. longipetala*. (B) Leaves of *E. longipetala*, leaf base truncate. (C) Leaves of *E. populnea*, leaf base cordate. (D) Whole plant of *E. populnea*. Photographs of *E. longipetala* and *E. populnea* were taken by Shuang Xiong and Shidong Wang in JinDian Park (25°5′5″N, 102°46′8″E) and Kunming Botanical Garden (102.71°E, 25.04°N), respectively.

The Hamamelidaceae is a family that connects the basal elements of the Rosidae and the “lower” Hamaelidae. Therefore, a better understanding of the phylogeny of the family is important for elucidating evolutionary patterns in the diversification of eudicots (Li et al. 1999b). *Exbucklandia* is one of the primitive genera of the family Hamamelidaceae (Li et al. 1999a, 1999b, Qiu et al. 1998, Ickert-Bond and Wen 2006, Xiang et al. 2019). The fossils of *Exbucklandia* could be dated to the early Cenozoic (Guo et al. 1984, Huang et al. 2017, Wu et al. 2009). *Exbucklandia* consists of only three extant species, which differ in morphological traits and distribution (Zhang et al. 2003, Huang et al. 2017). *Exbucklandia longipetala* H. T. Chang 1959 and *Exbucklandia populnea* R. W. Br. 1946, two evergreens within the genus (Figure 1), are mainly distributed in the subtropical evergreen broadleaved forests of Southeast Asia (Zhang et al. 2003, Huang et al. 2017). The two species are distinguished by the presence of a broad, cordate-based lamina or a slender, truncate-based lamina.

Previous studies on the phylogenetic relationships of Hamamelidaceae were based on chloroplast genome fragments and ITS sequences (Li et al. 1999a, 1999b, Xiang et al. 2019). In recent years, a phylogenetic tree of Hamamelidaceae was constructed using the complete chloroplast genomes, but the genus *Exbucklandia* was missing (Wang et al. 2022). Therefore, in this study, we chose to use complete chloroplast genomes to investigate the phylogenetic relationship of *Exbucklandia.* Here, the complete chloroplast genomes of *E. longipetala* and *E. populnea* are reported for the first time. We aim to provide insights into the chloroplast genome characterization and phylogenetic relationship of *E. longipetala* and *E. populnea*, which will provide potential genetic resources for further studies on the origin and evolution of Hamamelidaceae.

## Materials and methods

### Sample collection and DNA extraction

The fresh leaves of *E. longipetala* and *E. populnea* were collected from JinDian Park (25°5′5″N, 102°46′8″E) and Kunming Botanical Garden (102.71°E, 25.04°N), respectively, in Yunnan Province, China. The specimens were deposited in the herbarium of the School of Life Sciences, Yunnan Normal University (Kunming, China; Jianlin Hang, hjlynnu@163.com) under the accession numbers Y-1 (*E. longipetala*) and Y-27 (*E. populnea*). Total genomic DNA was extracted using a modified CTAB method (Porebski et al. 1997).

### Genome sequencing, assembly and annotation

The Illumina Hiseq X Ten platform was used to sequence the DNA of *E. longipetala* and *E. populnea* with a read length of 150 bp. The fastp v.0.23.2 software (Chen 2023) was then used to remove low quality sequences. The complete chloroplast genomes of the two species were assembled using NOVOPlasty v2.7.2 software (Dierckxsens et al. 2016). Annotation of the two chloroplast genomes was performed using GeSeq (Tillich et al. 2017), with *Rhodoleia championii* (Genbank accession number MK834325) as the reference genome. The results were then imported into Geneious v2023.0.1 software (Kearse et al. 2012) for manual adjustment. Finally, GenBank annotation files were generated and visualized using CPGView (http://www.1kmpg.cn/cpgview/) for chloroplast genome mapping.

### Phylogenetic analysis

A maximum likelihood (ML)-based phylogenetic tree was constructed using 20 chloroplast genome sequences, including 17 species from 13 genera of Hamamelidaceae, and 3 species of Altingiaceae as outgroups. The complete sequences of these genomes were further multiple aligned using MAFFT v7.47 software (Katoh and Standley 2013). The phylogenetic tree was then constructed using IQ-TREE v1.6.10 software (Nguyen et al. 2015) based on the substitution TVM + F+R2 best-fit model according to the Bayesian information criterion (Kalyaanamoorthy et al. 2017). Branch support was tested with 10,000 replicates using ultrafast bootstrap (UFBoot) (Hoang et al. 2018) and SH-like approximate likelihood ratio test (SH-aLRT) (Guindon et al. 2010). The phylogenetic tree was visualized using Figtree (https://github.com/rambaut/figtree).

## Results

### Characteristics of chloroplast genome

We sequenced the whole genome sequences of *E. longipetala* and *E. populnea* using Illumina Hiseq X Ten platform with an average coverage of 2574.2 and 1097.5, respectively (Supplementary Figure S1). As a result, a total of 44,443,964 and 30,474,644 filtered reads were obtained for *E. longipetala* and *E. populnea,* respectively (Supplementary Figure S2). The annotated chloroplast genomes of *E. longipetala* and *E. populnea* were submitted to NCBI Genebank under the accession numbers PP209140 and ON584550.

**Figure 2.**
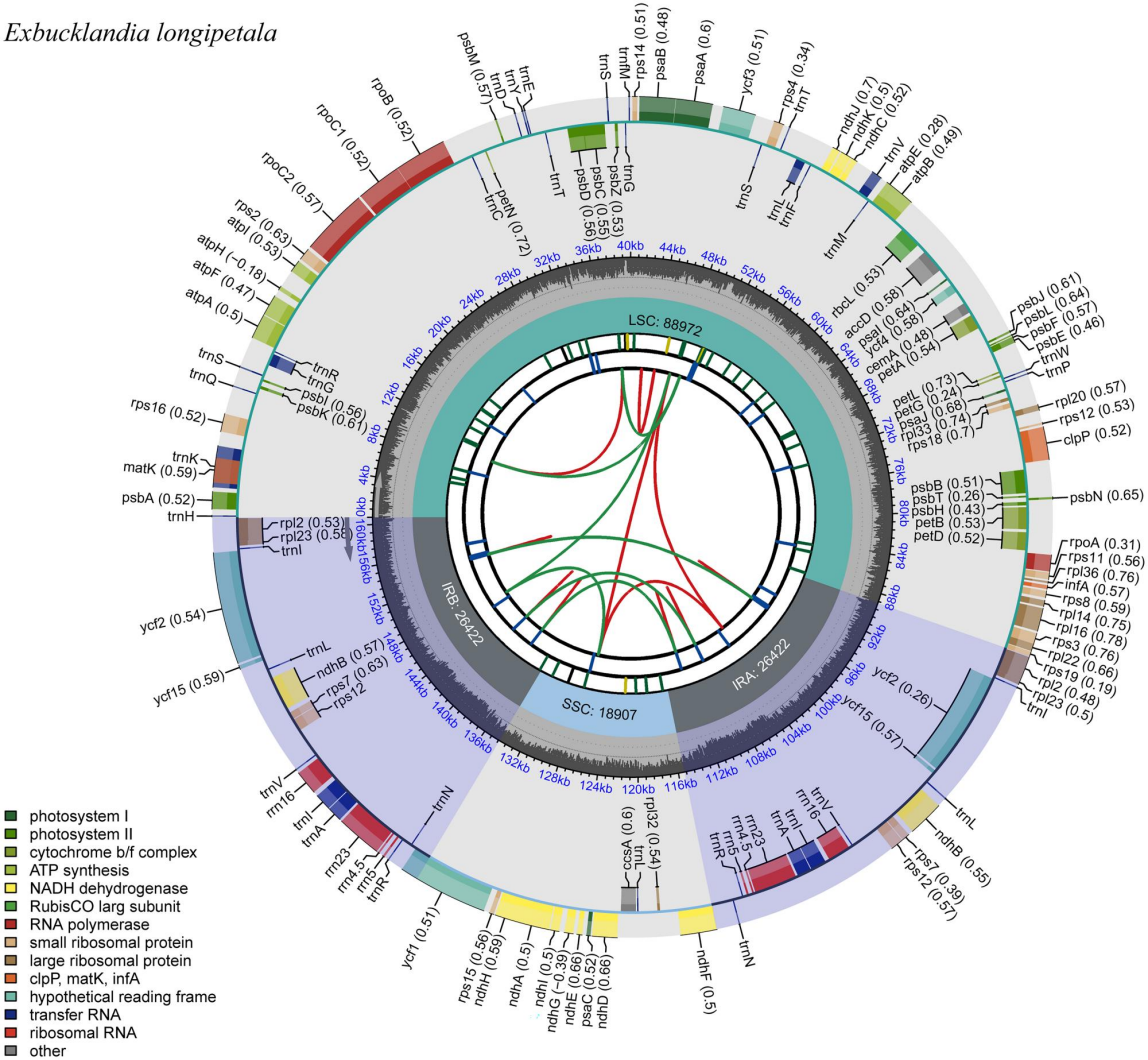
Genomic map of overall features of the chloroplast genome of *E. longipetala* by CPGview. The map contains six tracks from the center outward. The first track represents the dispersed repeats, the red arcs represent the direct repeats and the green arcs represent the palindromic repeats. The second track is a long tandem repeat marked by short blue bars. The third track shows the short tandem repeats or microsatellite sequences as short bars with different colors. The small single-copy (SSC), inverted repeat (IRs), and large single-copy (LSC) regions are shown on the fourth track. The fifth track is the GC content of the chloroplast genome. The last track is coding genes categorized according to function. The transcription directions for the inner and outer genes are clockwise and anticlockwise, respectively.

The complete chloroplast genomes of *E. longipetala* and *E. populnea* were closed circular molecules of 160,723 bp and 160,744 bp in length, respectively, with the same total GC content of 38.1% (Figure 2 and Supplementary Figure S3). Both chloroplast genomes contained typical quadripartite structures with a large single-copy (LSC) region of 88,972 bp (*E. longipetala*) and 88,989 bp (*E. populnea*), a small single-copy (SSC) region of 18,907 bp (*E. longipetala*) and 18,911 bp (*E. populnea*), and a pair of inverted repeat (IR) regions both of 26,422 bp each.

Moreover, both chloroplast genomes of *E. longipetala* and *E. populnea* contained the same genes, which were composed of 87 protein-coding genes, 37 tRNA genes, and 8 rRNA genes, including 80 unique protein-coding genes, 30 unique tRNA genes, and 4 unique rRNA genes. Furthermore, the 114 unique genes could be divided into four types according to their functions: photosynthesis, self-replication, other genes, and genes of unknown function (Supplementary Table S1). In total, one trans-splicing gene (*rps*12) and 11 cis-splicing genes (*rpl*2, *ndh*B, *ndh*A, *rpl*16, *pet*D, *pet*B, *clp*P, *ycf*3, *rpo*C1, *atp*F, *rps*16) were identified (Supplementary Figure S4).

### Phylogenetic relationship

**Figure 3.**
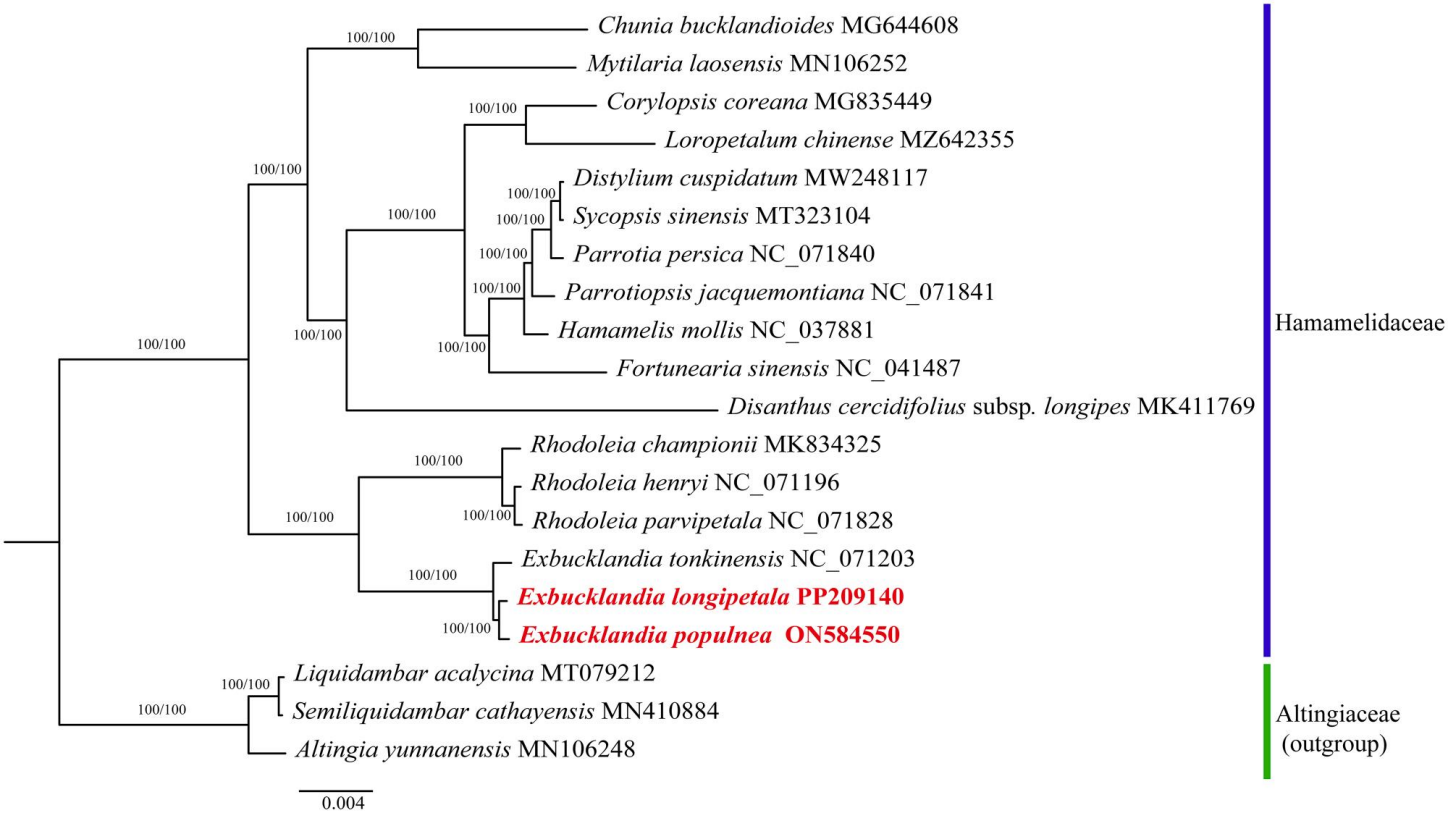
A phylogenetic tree was constructed using the maximum-likelihood method based on complete chloroplast sequences from 17 Hamamelidaceae species and 3 Altingiaceae species as outgroups. Branch support values were reported as UFBoot/SH-aLRT. The bolded red font represented the chloroplast genomes of *E. longipetala* and *E. populnea* in this study. The following sequences were used: *Hamamelis mollis* NC_037881, *parrotiopsis jacquemontiana* NC_071841, *distylium cuspidatum* MW248117 (Dong et al. 2021), *sycopsis sinensis* MT323104 (Peng et al. 2020), *parrotia persica* NC_071840, *fortunearia sinensis* NC_041487, *corylopsis coreana* MG835449 (Choi et al. 2019), *loropetalum chinense* MZ642355 (Wang et al. 2022), *disanthus cercidifolius* subsp. *longipes* MK411769 (Yu et al. 2019), *Chunia bucklandioides* MG644608 (Zhou et al. 2019), *Mytilaria laosensis* MN106252 (Wang et al. 2019), *Rhodoleia championii* MK834325 (Li et al. 2019), *Rhodoleia henryi* NC_071196, *Rhodoleia parvipetala* NC_071828, *Exbucklandia tonkinensis* NC_071203, *liquidambar acalycina* MT079212 (Lai et al. 2020), *semiliquidambar cathayensis* MN410884 (Shi et al. 2019), *altingia yunnanensis* MN106248 (Qiu et al. 2020).

To further investigate the phylogenetic relationship of *E. longipetala* and *E. populnea* with other Hamamelidaceae plants, a total of 18 additional chloroplast genomes were obtained from the NCBI database. The maximum likelihood phylogenetic tree was composed of 20 species divided into two major clades (Figure 3). The phylogenetic analysis showed that the species of Hamamelidaceae and three outgroup species each form a highly supported monophyletic group. The Hamamelidaceae branch was divided into two clades, and species of the same genus tended to cluster together. *Exbucklandia* and *Rhodoleia* formed a robust monophyletic clade and be sister genera to each other, while the other Hamamelidaceae species clustered in a relatively large branch. Three species of *Exbucklandia* formed a strongly monophyletic clade. *E. longipetala* and *E. populnea* were sister species to each other.

## Discussion and conclusion

In this study, the complete chloroplast genomes of *E. longipetala* and *E. populnea* were reported for the first time. Both chloroplast genomes contained 114 unique genes, including 80 protein-coding genes, 30 tRNA genes, and 4 rRNA genes. The phylogenetic analysis showed that three species of *Exbucklandia* cluster together and form sister groups with *Rhodoleia*, which is supported by the phylogeny based on *mat*K data (Li et al. 1999b). The phylogenetic tree based on the ITS DNA suggested that *Exbucklandia, Rhodoleia*, and *Mytilaria* formed a clade (Li et al. 1999a). However, in our study, the genus *Mytilaria* was sister to *Chunia*, rather than nested in the *Exbucklandia* and *Rhodoleia* clade. As *Chunia* is absent from the ITS-based phylogenetic tree, the relationship between *Chunia* and *Mytilaria* needs further investigation. Our results also showed that *Exbucklandia* is the basal taxon within Hamamelidaceae (Ickert-Bond and Wen 2006, Li et al. 1999a). Furthermore, the results suggest that *E. longipetala* is closely related to *E. populnea* and may be the most primitive of the extant species of *Exbucklandia*. Nevertheless, the chloroplast genomes will provide useful genetic resources for the studies on the classification, phylogeny, and evolutionary history of the Hamamelidaceae.

## Supplementary Material

Supplementary.docx

## Data Availability

The genome sequence data that support the findings of this study are openly available in GenBank of NCBI at [https://www.ncbi.nlm.nih.gov/] under accession no. PP209140 (*E. longipetala*) and ON584550 (*E. populnea*). The associated BioProject, SRA, and Bio-Sample numbers for *E. longipetala* are PRJNA1068818, SRR27733264, and SAMN39604683, respectively, while those for *E. populnea* are PRJNA841841, SRR19391822, and SAMN28626150, respectively.
