# Supplementary material for: The complete chloroplast genomes and phylogenetic analysis of Exbucklandia longipetala and Exbucklandia populnea (Hamamelidaceae)
Source: Mitochondrial DNA B Resour. 2024 Sep 30;9(10):1279–84. doi: 10.1080/23802359.2024.2406933 (PMC11443542; doi:10.1080/23802359.2024.2406933)
Supplement: Supplementary.docx [file TMDN_A_2406933_SM0854.docx]

**
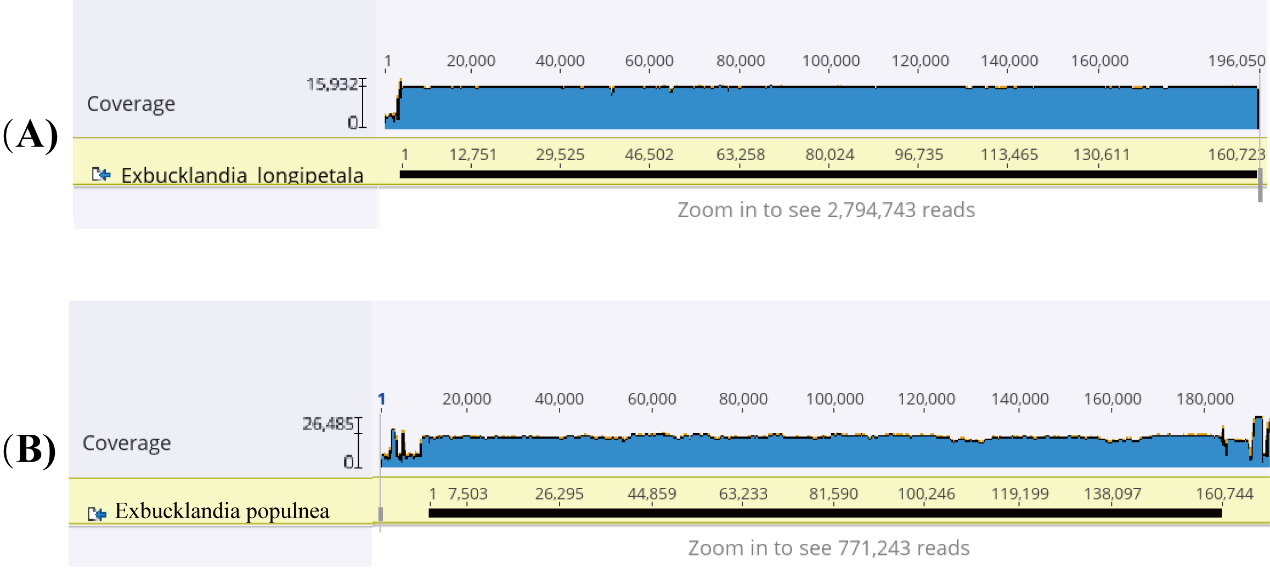
**

**Figure S1**. The coverage figure of the complete chloroplast genomes of *E. longipetala* (A) and *E. populnea* (B)

**
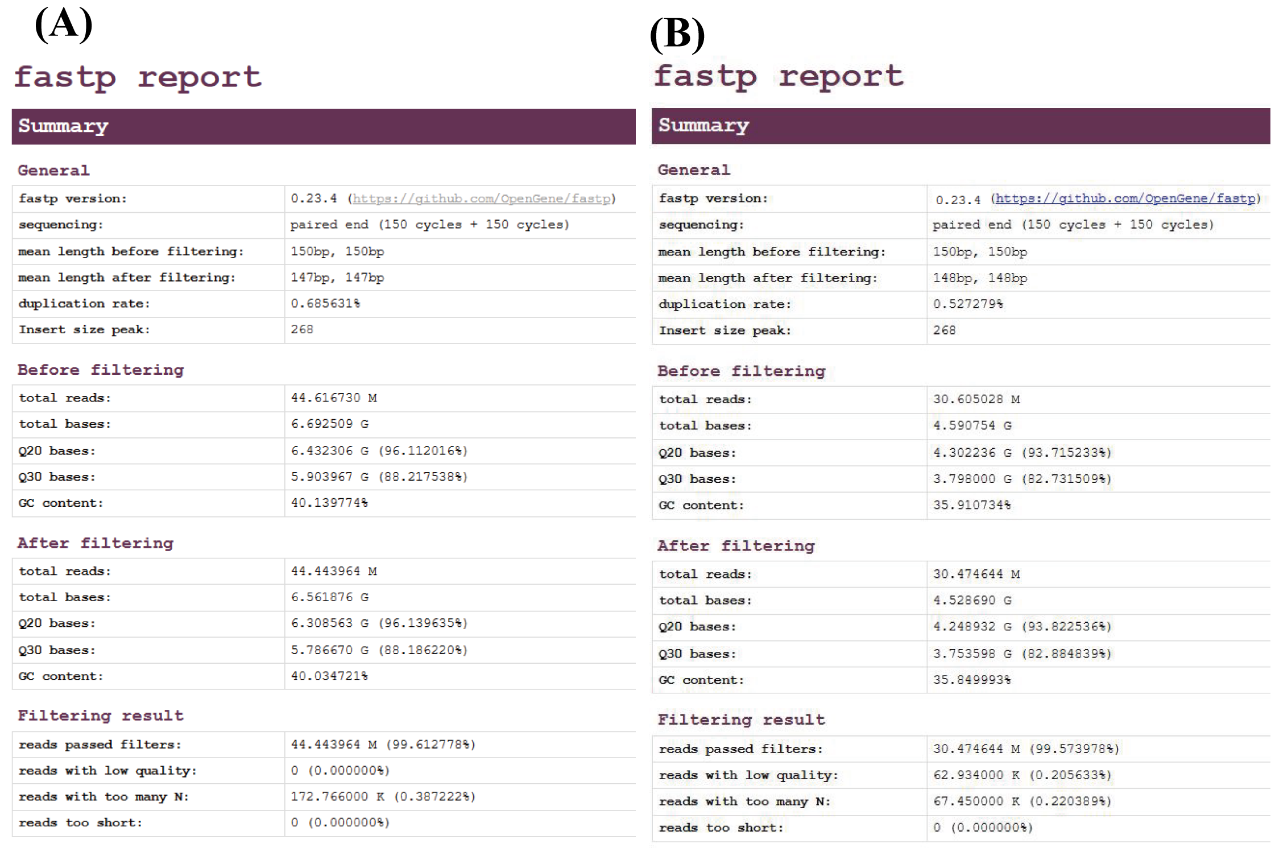
**

**Figure S2**. The QC data of the complete chloroplast genomes of *E. longipetala* (A) and *E. populnea* (B)

**
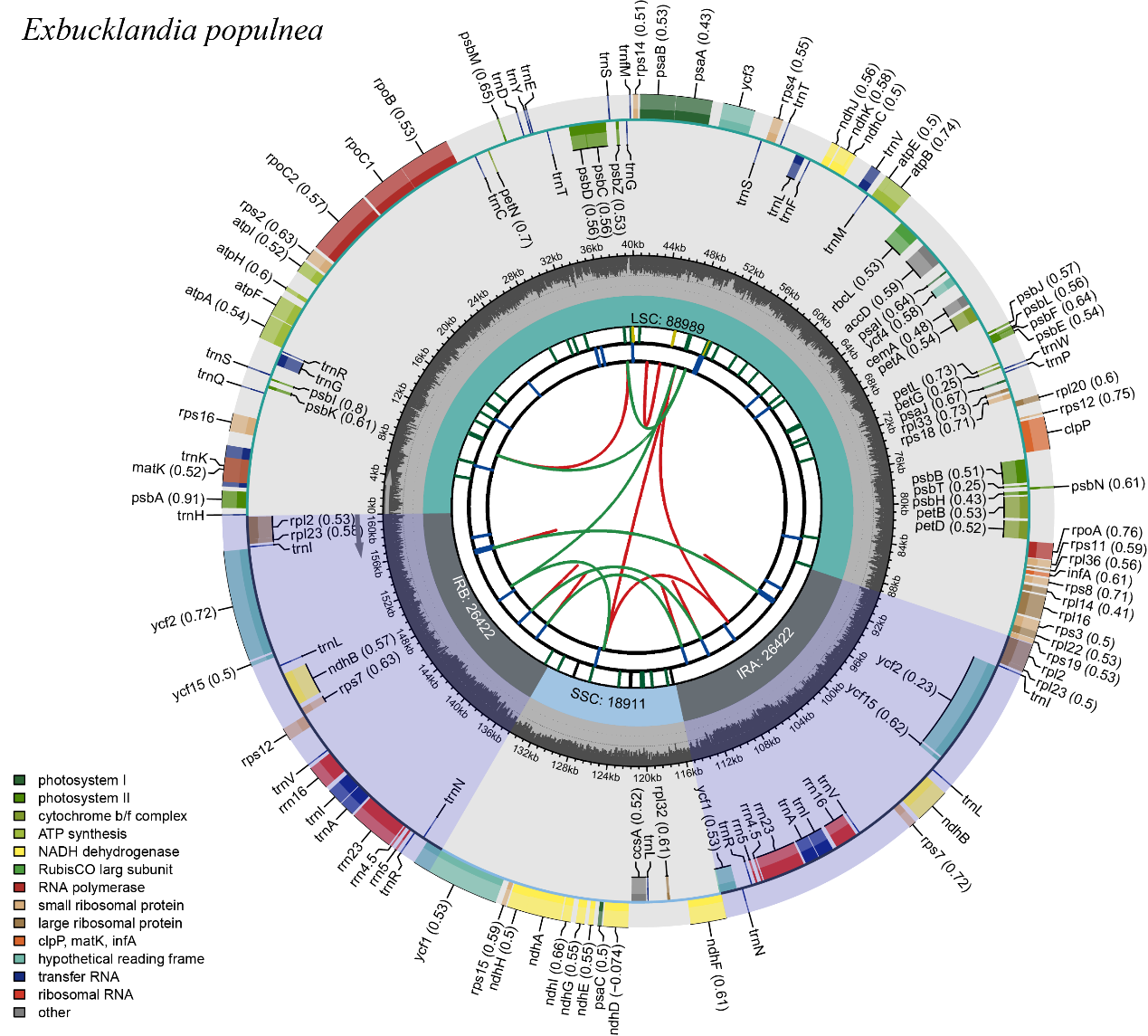
Figure S3**. Genomic map of overall features of the chloroplast genomes of *E. populnea* by CPGview. The map contains six tracks from the center outward. The first track represents the dispersed repeats, the red arcs represent the direct repeats and the green arcs represent the palindromic repeats. The second track is a long tandem repeat marked by short blue bars. The third track shows the short tandem repeats or microsatellite sequences as short bars with different colors. The small single-copy (SSC), inverted repeat (IRs), and large single-copy (LSC) regions are shown on the fourth track. The fifth track is the GC content of the chloroplast genome. The last track is coding genes categorized according to function. The transcription directions for the inner and outer genes are clockwise and anticlockwise, respectively.

**Table S1** Gene annotation of the chloroplast genomes of *E.longipetala* and *E. populnea*

| Category of genes | Group of genes | Name of genes |
| --- | --- | --- |
| Genes for photo-synthesis | Subunits of ATP synthase | *atp*A, *atp*B, *atp*E, *atp*F, *atp*H, *atp* I |
|  | Subunits of photosystem II | *psb*A, *psb*B, *psb*C, *psb*D, *psb*E, *psb*F, *psb*H, *psb*I, *psb*J, *psb*K, *psb*L, *psb*M, *psb*N, *psb*T, *psb*Z, *ycf*3 |
|  | Subunits of NADH-dehydrogenase | *ndh*A, *ndh*B^*^, *ndh*C, *ndh*D, *ndh*E, *ndh*F, *ndh*G, *ndh*H, *ndh*I, *ndh*J, *ndh*K |
|  | Subunits of cytochrome b/f complex | *pet*A, *pet*B, *pet*D, *pet*G, *pet*L, *pet*N |
|  | Subunits of photosystem I | *psa*A, *psa*B, *psa*C, *psa*I, *psa*J |
|  | Subunits of rubisco | *rbc*L |
| Self-replication genes | Ribosomal RNA genes | *rrn*4.5^*^, *rrn*5^*^, *rrn*16^*^, *rrn*23^*^ |
|  | Large subunit of ribosome | *rpl*2^*^, *rpl*14, *rpl*16, *rpl*20, *rpl*22, *rpl*23^*^, *rpl*32, *rpl*33, *rpl*36 |
|  | DNA dependent RNA polymerase | *rpo*A, *rpo*B, *rpo*C1, *rpo*C2 |
|  | Small subunit of ribosome | *rps*2, *rps*3, *rps*4, *rps*7^*^, *rps*8, *rps*11, *rps*12^*^, *rps*14, *rps* 15, *rps*16, *rps*18, *rps*19 |
|  | Transfer RNAs | *trnA-UGC*, trnC-GCA, trnD-GUC, trnE-UUC, trnF-GAA, trnfM-CAU, trnG, trnG-UCC, trnH-GUG, trnI-CAU*, trnI-GAU*, trnK-UUU, trnL-CAA*, trnL-UAA, trnL-UAG, trnM-CAU, trnN-GUU*, trnP-UGG, trnQ-UUG, trnR-ACG*, trnR-UCU, trnS-GCU, trnS-GGA, trnS-UGA, trnT-GGU, trnT-UGU, trnV-GAC*, trnV-UAC, trnW-CCA, trnY-GUA* |
| Other genes | Subunit of Acetyl-CoA-carboxylase  c-type cytochrome synthesis gene  Envelop membrane protein  ATP-dependent protease subunit P  Translational initiation factor  Maturase | *acc*D  *ccs*A  *cem*A  *clp*P  *inf*A  *mat*K |
| Unknown functional genes | Conserved open reading frames | *ycf*1, *ycf*2^*^, *ycf*4, *ycf*15^*^ |

Note: *Two copies

**
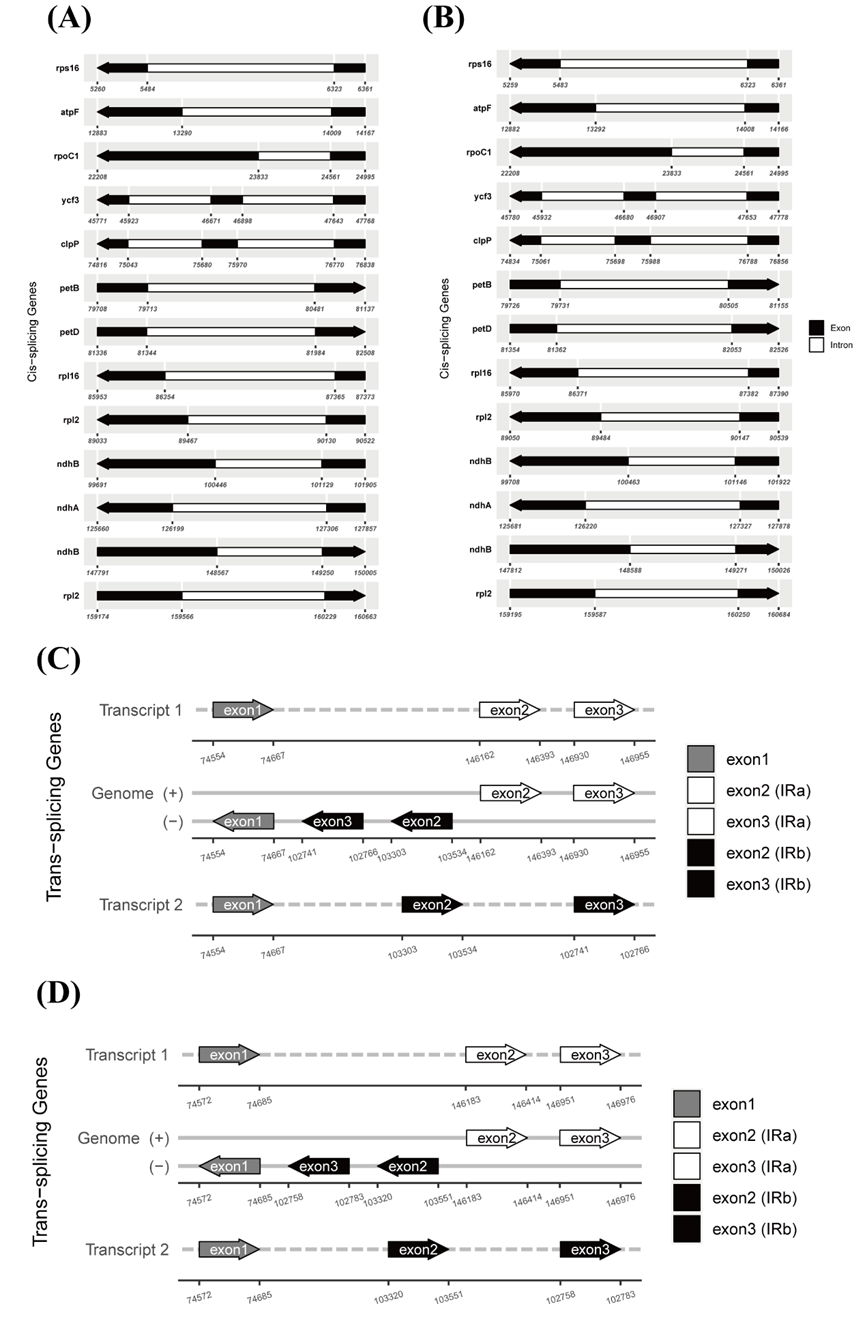
Figure S4**. Schematic map of the cis and trans-splicing genes in the chloroplast genomes of *E. longipetala* (A) (C) and *E. populnea* (B) (D)
